# Auditory distraction transmitted by a cochlear implant alters allocation of attentional resources

**DOI:** 10.3389/fnins.2015.00068

**Published:** 2015-03-05

**Authors:** Mareike Finke, Pascale Sandmann, Bruno Kopp, Thomas Lenarz, Andreas Büchner

**Affiliations:** ^1^Cluster of Excellence “Hearing4all”Hannover, Germany; ^2^Department of Otolaryngology, Hannover Medical SchoolHannover, Germany; ^3^Department of Neurology, Hannover Medical SchoolHannover, Germany

**Keywords:** cochlear implants, resource capture, distraction, novelty, event-related potentials, N1, P3, CIRC model

## Abstract

Cochlear implants (CIs) are auditory prostheses which restore hearing via electrical stimulation of the auditory nerve. The successful adaptation of auditory cognition to the CI input depends to a substantial degree on individual factors. We pursued an electrophysiological approach toward an analysis of cortical responses that reflect perceptual processing stages and higher-level responses to CI input. Performance and event-related potentials on two cross-modal discrimination-following-distraction (DFD) tasks from CI users and normal-hearing (NH) individuals were compared. The visual-auditory distraction task combined visual distraction with following auditory discrimination performance. Here, we observed similar cortical responses to visual distractors (Novelty-N2) and slowed, less accurate auditory discrimination performance in CI users when compared to NH individuals. Conversely, the auditory-visual distraction task was used to combine auditory distraction with visual discrimination performance. In this task we found attenuated cortical responses to auditory distractors (Novelty-P3), slowed visual discrimination performance, and attenuated cortical P3-responses to visual targets in CI users compared to NH individuals. These results suggest that CI users process auditory distractors differently than NH individuals and that the presence of auditory CI input has an adverse effect on the processing of visual targets and the visual discrimination ability in implanted individuals. We propose that this attenuation of the visual modality occurs through the allocation of neural resources to the CI input.

## Introduction

Cochlear implants (CIs) bypass a non-functional inner ear by a direct electrical stimulation of the auditory nerve. Compared to normal acoustic hearing, sounds transmitted through the CI are degraded (Drennan and Rubinstein, 2008). Following CI implantation, the (re-)acquisition of speech intelligibility is considered as a desirable result of CI rehabilitation (Krueger et al., 2008). Until now, it remains unknown how auditory cognition adapts to the degraded input from the CI. Many CI users, however, experience difficulties in more challenging listening tasks such as speech intelligibility in noise (Wilson and Dorman, 2008) and it is well recognized by the field that the individual CI outcomes is difficult to predict (Peterson et al., 2010). Recently, it has been discussed that higher-order, central resources might play a role in CI rehabilitation (Pichora-Fuller and Singh, 2006; Pichora-Fuller, 2006, 2008; Humes, 2007).

Cognitive abilities are strictly limited by quantitative constraints on processing capacity (Cherry, 1953; Broadbent, 1958; Deutsch and Deutsch, 1963; Treisman, 1964). The limitation of attentional resources in particular can be demonstrated nicely with the so-called attentional blink (AB). This refers to the phenomenon that humans often fail to detect a second target (T2) if it is presented between 200 and 500 ms after a first target (T1) within rapid series of stimuli (Raymond et al., 1992). Vogel et al. (1998) recorded ERPs and showed that the failure to detect T2 in temporal vicinity of T1 is caused by lacking attentional rather than perceptual resources.

To date, a consensus with regard to the unity (i.e., a central amodal resource) or diversity (i.e., multiple modality-specific resources) of attentional resources has not been achieved. For example, Kahneman's model assumes a limited-capacity central resource plus a separate unit which is capable of distributing various parts of the central resource over specific task demands (Kahneman, 1973). If we apply this idea to CI-mediated hearing and listening, the presence of CI input might trigger the allocation of limited-capacity central resources for attentional processing of this degraded input. Importantly, these central resources would not be allocated to auditory input in normal-hearing (NH) listeners.

This idea bears some similarities with the ease of language understanding (ELU) model (Rönnberg et al., 2013). According to the ELU model, auditory input matches sufficient numbers of attributes stored in long-term memory under ideal conditions, and listening proceeds rapidly and automatically. Whenever there is a mismatch, as in the case of suboptimal listening conditions, speech understanding is supported by additional explicit processing (Rönnberg et al., 2013). Importantly, the ELU model proposes that the explicit listening effort is triggered by mismatches between attributes of the current input and stored attributes. The present study does not investigate linguistic processes as such. However, the allocation of resources toward auditory input is not restricted to complex information like speech. Even if the auditory input is task-irrelevant, it can influence task performance. The common ground between the mentioned aspect of the ELU model and our idea is that in adverse listening conditions additional attentional resources are needed to process the auditory input which might in turn lack for other tasks or distract from the actual task. Listening via a CI is having adverse listening conditions at any time. Therefore, we assume that auditory input via the CI might capture central resources under all circumstances, due to degraded signal quality.

The present study aims to the better understanding of how the cortical allocation of attentional resources differs between CI users and NH participants. We used event-related brain potentials (ERPs) to compare perceptual and post-perceptual cortical responses between CI users and NH participants. Perceptual neural processing expresses itself in early ERP components, whereas post-perceptual neural processing is reflected in later ERP components (Vogel et al., 1998; Escera et al., 2000; Näätänen et al., 2005; Polich, 2007; Cortiñas et al., 2008; SanMiguel et al., 2008; Schomaker and Meeter, 2014).

The so-called orienting response toward a novel *task-irrelevant auditory* distractor is reflected in an ERP component called novelty-P3 (Escera et al., 2000). Similarly, in the *visual* domain, task-irrelevant processing seems to be reflected by a frontal N2 component (Schomaker and Meeter, 2014). In terms of *task-relevant* information processing, the P3 response (also called P3b) has been identified to auditory as well as visual targets. It reflects the allocation of attention that is required to evaluate a task-relevant event and to update working memory accordingly (Donchin and Coles, 1988, 1998; Verleger, 1997, 2008; Vogel et al., 1998; Kok, 2001; Polich, 2007). For simplicity, we will use the term P3 to refer to the P3b in this article. All three mentioned ERP components (novelty-P3, novelty-N2, P3) are associated with post-perceptual stimulus processing, whereas earlier ERP components (P1, N1, P2) reflect mainly perceptual processing of the respective stimuli.

The perceptual and post-perceptual processes of task-irrelevant distractors and task-relevant targets can be investigated by means of the cross-modal discrimination-following-distraction (DFD) paradigm. This paradigm classically uses auditory distractors which are followed by a visual target, and allows the examination of the orienting response toward task-irrelevant distractors and the resource capture they induce (Escera et al., 1998, 2000; SanMiguel et al., 2008). In this study we used the described cross-modal DFD paradigm to examine the allocation of attentional resources toward task-relevant and novel stimuli in CI users and NH listeners. Importantly, we used two variants of this DFD paradigm: On each experimental trial, the *visual-auditory DFD* combined visual distraction with immediately following auditory discrimination performance, while auditory distraction preceded visual discrimination performance on the *auditory-visual DFD* (Escera et al., 1998). The two versions of the DFD paradigm enabled us to examine the processing of auditory or visual distractors and the processing of the subsequent (cross-modal) visual or auditory targets.

Similar to previous studies, we used three different types of distractors (Escera et al., 1998). Standard and deviant distractors were repetitive tones which occurred with high or low probability (80% standard distractors, 10% deviant distractors). Novel distractors constituted the third distractor type and occurred with the same probability as deviant sounds (10%). Novel distractors were different environmental sounds and were presented only once. Among the task-irrelevant auditory distractors, a novelty-P3 is elicited only by novel distractors but not by repetitive distractors (Escera et al., 1998; Cortiñas et al., 2008; SanMiguel et al., 2008). This peak reflects the orienting response toward the novel and therefore unexpected event (Escera et al., 1998, 2000). The comparison of cortical and behavioral responses elicited by the three different distractor types allowed us to study the particular effect of repeated (standard, deviant distractors) and novel distractors on resource capturing. As previously mentioned, the ELU model proposes that auditory input is only processed explicitly when auditory input does not match sufficient numbers of attributes stored in long-term memory (Rönnberg et al., 2013). In the present study, standard distractors—but not novel distractors—occured in a repetitive manner and their attributes could thus be stored in long-term memory after some repetitions. Consequently, the comparison of responses to deviant and novel stimuli in CI users allowed us to address the question of whether the proposed resource capture is specifically induced by novel CI input, or alternatively by any CI input, irrespective of its novelty or its familiarity.

Similar to the results from NH listeners, we predicted an orienting response and behavioral costs induced by auditory and visual distractors compared to standard distractors in both the NH listeners and the CI users (Escera et al., 1998, 2000; SanMiguel et al., 2008; Schomaker and Meeter, 2014). Moreover, we hypothesized that post-perceptual ERPs in response to auditory distractors (novelty-P3) differ between CI users and NH participants. According to our hypothesis of altered attentional resource allocation in CI users, visual targets (P3) that followed auditory distractors should be attenuated in the CI users when compared with NH listeners. The latter prediction is a straightforward consequence of our assumption that limited-capacity central resources are allocated to the attentional processing of the degraded auditory CI input. In contrast, post-perceptual ERPs in response to visual distractors (novelty-N2) should be unaffected in CI users, because the visual input is comparable in the two groups.

## Materials and methods

### Participants

Twelve CI users (9 male, 3 female; 10 right-handed) and 12 normal-hearing controls (6 male, 6 female; 10 right-handed) participated in the present study. Because of the considerable age range across CI users (mean age and standard deviation: 43.67 ± 15.36 years; range 20–63 years), each CI user was matched with a normal-hearing participant for age (mean age and standard deviation (SD): 43.33 ± 15.56 years; range 19–64 years). Consequently, groups did not differ in age [*t*_(22)_ = 0.053, *p* = 0.958]. All participants had normal or corrected-to-normal vision and no history of neurologic or psychiatric illness. CI users were invited to participate in the study when they had a minimum speech understanding of 20% in the Hochmair-Schulz-Moser (HSM) sentence test in noise (10 dB signal-to-noise ratio; SNR) for their last test visit at our clinic (Hochmair-Desoyer et al., 1997). Performance in the HSM sentence test in quiet was 97% on average (SD ± 4.4%) and 48% on average (SD ± 20.7%) for the HSM in noise. Seven CI users were unilaterally implanted and 5 bilaterally. All participants had been using their CI for at least 12 months before the experiment and none of the CI users used sign language to communicate. Table 1 provides the details about the CI system, the speech processor and the clinical history of each CI user. In case of bilateral implantation the ear with better test scores was selected. All CI users received the auditory stimulation via an audio cable. Each NH participant was tested at the same ear as their match in the CI users group. Their normal hearing was assured by pure tone audiometry (250–4000 Hz) in the tested ear. In total three right ears and nine left ears were tested in each group. Participants gave informed written consent before the experiment. The experimental protocol was approved by the Ethical Committee of the Hannover Medical School and was in accordance with the Code of Ethics of the World Medical Association (Declaration of Helsinki).

**Table 1:** **Participant demographics of the cochlear implant group**.

**Ci user**	**Age (y)**	**Tested ear**	**Implant**	**Speech processor**	**Etiology**	**CI use (m)**	**2nd CI use (m)**	HSM^*^ (10 dB SNR)	HSM^*^ (quiet)
1	34	Left	Medel Pulsar	Opus 2	Progressive	88	70	53	99
2	63	Right	Medel concerto	Opus 2	Progressive	48	69.8	70	100
3	26	Left	Nucleus RE-24	CP 810	Sudden deafness	24	–	20	97
4	58	Left	Nucleus CI422	CP 810	Progressive	36	–	15	85
5	50	Left	Nucleus CI512	CP 810	Progressive	46	70	29	100
6	46	Left	Nucleus R-24 CS	CP 810	Progressive	24	0.25	29	94
7	32	Left	Nucleus CI512	CP 810	Progressive	33	–	44	98
8	20	Left	Nucleus R-24 CS	CP 810	Sudden deafness	149	24	81	100
9	55	Left	Nucleus RE-24	CP 810	Sudden deafness	12	–	50	100
10	26	Right	Nucleus 22	Freedom	Sudden deafness	23	0	60	100
11	63	Right	AB HiRes90K	Harmony	Progressive	12	–	30	95
12	51	Left	Nucleus CI512	CP 810	Progressive	24	–	27	94

* *Measured as percent correct in the sentence Test HSM*.

### Task and procedure

**Figure 1:**
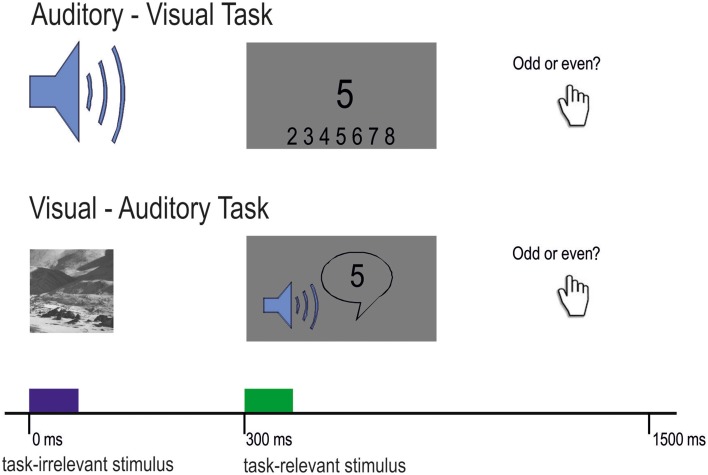
**Experimental design of the auditory-visual task (upper part) and the visual-auditory task (lower part)**. Participants had to decide in both conditions whether the presented number (visual or auditory) was odd or even. Task-irrelevant stimuli were randomized with 80% standard, 10% deviant, and 10% novel stimuli.

Participants were tested with two cross-modal DFD tasks. The first task was the well-established *auditory–visual distraction task* developed by Escera et al. (1998). Consistent with previous studies, we presented stimuli in pairs of a task-irrelevant auditory distractor and a visual task-relevant target. Auditory distractors were either standard tones (80%) or deviant tones (10%) and novel sounds (10%). One trial consisted of a 200 ms auditory distractor followed by the visual target after 300 ms (Figure 1).

The standard and deviant distractors were sinusoidal tones of 200 ms duration including 10 ms rise and fall times with the respective frequencies of 600 and 756 Hz (4 semitones apart). In the original version of the paradigm (Escera et al., 1998) the two tones were 600 and 700 Hz. We extended this difference between tones for the present study due to a limited frequency resolution in CI users. Novel distractors were different environmental sounds, such as those produced by a drill, hammer, motor or a telephone (Escera et al., 1998). From the original set (Escera et al., 1998), we used those novel sounds which have rated as most identifiable in a previous study (Escera and Corral, 2003). They were digitally recorded, low-pass filtered at 10,000 Hz and edited to have a duration of 200 ms, including rise and fall times of 10 ms and an intensity maximum of 70–80 dB SPL (sound pressure level). Each novel stimulus was presented exactly once throughout the experiment. Distractors were presented in randomized order with the constraint that both the deviant and the novel distractors were preceded by at least two standard distractors. All auditory stimuli were presented monaurally via insert-earphones at 70 dB SPL in normal-hearing participants or via an audio cable connected to the CI speech processor. Loudness scaling, a method usually used in clinical context was used to adjust loudness in CI users to a moderate level. The visual targets were digits from 2 to 9 presented randomly in the center of the screen for 300 ms. Participants were comfortably seated in a dimly lit, electrically and acoustically shielded booth. Due to the clinical background of the study, we enlarged the original inter-trial interval (ITI) of 1200–1500 ms as it has been done previously with a different group of patients (Escera et al., 1998; Cortiñas et al., 2008).

The second task was a *visual-auditory distraction task*. For this task, we reversed the modalities of the original paradigm, meaning that the task-irrelevant distractors were visual and the target stimuli were auditory (see Figure 1). All other parameters were kept constant. Visual distractors were presented for 200 ms and were followed by the task-relevant auditory stimulus. Standard and deviant images were Gabor patches [spatial frequency: 4 cycles per degree (cpd)] presented on a gray background and tilted 45° either to the left or to the right. Novel images were taken from the Psychological Image Collection at Stirling (PICS; http://pics.psych.stir.ac.uk/), which have been used in previous research (Bunzeck et al., 2012, 2014). In order to avoid influences of emotion or face-specific processing on ERPs, images did not contain humans in the foreground or potentially threatening content but landscape or street scenes (Kanwisher et al., 1997; Eimer and Holmes, 2002). In order to keep luminance equal across images, all images (including the two Gabor patches) were normalized using the SHINE Toolbox (Willenbockel et al., 2010) for Matlab (Mathworks). Auditory targets were spoken digits from 2 to 9 with a duration of 300 ms, and were adapted from original items of the Freiburg speech test (Hahlbrock, 1953).

For both tasks, participants were instructed to fixate the dot in the middle of the screen and to identify the presented numbers as odd or even with their left (odd) or right (even) thumb. The participants were asked to respond as quickly and correctly as possible. No feedback on single trials was given to the participants. Prior to the experimental blocks, participants completed a short training. After the training block it was ensured that CI users could distinguish standard sounds from deviant sounds and that novel sounds did sound differently from each other. In total, participants completed 800 trials (640 standard, 80 deviant, 80 novel trials) presented in 4 blocks of 200 trials for each task. The duration of the experiment was 40 min plus recovery time between the experimental blocks. The tone mapping of standard and deviant tones was reversed in half of the blocks: In two blocks the 600 Hz and the 756 Hz tone served as the standard, respectively (auditory-visual DFD). Likewise, the left tilted Gabor patch served as a standard or the deviant in 50% of the trials, respectively (visual-auditory DFD).

### Data recording and analysis

#### Behavioral data

For behavioral analysis, a correct trial was defined as a correct button press that occurred between 100 and 1200 ms from target onset (visually presented or spoken numbers). The individual mean of a participants' response time (RT) relative to target-onset was computed for correct trials only. RTs as well as hit rates (HRs) were analyzed using two repeated measures 2 × 3 × 2 ANOVAs with the between-subjects factor Group (CI, NH) and the two within-subjects factors Modality (auditory-visual DFD, visual-auditory DFD) and Distractor Type (standard, deviant, novel sounds or images).

#### Electrophysiological data

EEG was continuously recorded by a SynAmps amplifier (Compumedics, Neuroscan) from 78 scalp electrodes using a 128-channel Quik-Cap (Neuroscan). Two additional electrodes were placed at the left and right mastoids. For encephalic electrode locations of the Neuroscan system see www.neuroscan.com. The common reference electrode for these channels was placed at the tip of the nose. Horizontal and vertical electrooculography (EOG) was recorded bipolarly from four electrodes placed at the outer canthi of both eyes as well as above and below the right eye. The EEG was amplified and digitized at 1 kHz, and impedances were kept below 20 kOhms during the whole recording session.

EEG data was processed offline with a band pass filter from 0.1 to 30 Hz and averaged over 1400 ms epochs including a 200 ms pre-stimulus baseline. EOG correction was performed using a covariance algorithm (Semlitsch et al., 1986) and Principle Component Analysis (PCA) was used to reduce artifacts induced by the CI (CURRY Scan 7 Neuroimaging Suite, Neuroscan). This well-established multivariate data technique transforms the data into an orthogonal coordinate system. The first coordinate lies along the direction of most variance in the data. Subsequent coordinates explain as much as possible of the remaining variance. It can be assumed that the coordinate with most variation within the EEG signal contains the CI induced artifacts. The difference between CI artifact and EEG signal is quite prominent. PCA decomposition of CI artifact contaminated EEG data was performed and artifact related principal components were removed from the EEG. Previous studies have shown that short-lasting artifacts induced by non-cortical sources (like blinks or magnetic/electric pulses) can be reliably removed using PCA (Dien, 2010; Ter Braack et al., 2013). Trials containing signals which exceeded ± 100 μV in any of the scalp electrodes were excluded from further analyses. Missing channels located in the area of the speech processor and the transmitter coil were excluded for further analyses. The same channels were also excluded in the NH participants.

Only those standard trials which were followed by a deviant or novel trial were included in the analyses. For this reason, prior to each deviant or novel stimulus there were at least two the standard sounds. This ensures that there is a representation of the regular aspect of the standard stimulus in the sensory memory in these trials (Näätänen et al., 2005). All signal processing was carried out by means of CURRY Scan 7 Neuroimaging Suite (Neuroscan).

For the *visual-auditory DFD*, the P2 peak amplitude and latency elicited by the visual distractors was analyzed for each of the three distractor types in the time-window 170–240 ms after stimulus onset at I1, OI1, OI2, and I2. Consistent with the procedures used for the auditory-visual DFD, we computed difference waveforms to analyze the novelty detection in the visual domain (Schomaker and Meeter, 2014). The “novelty-N2” was computed by subtracting the individual ERPs elicited by the standard distractor from those elicited by novel distractors. Peak amplitude and latency of the novelty-N2 component were determined in the 180–330 ms time-window at Fz, FCz, and Cz.

Beyond that, the P3 component elicited by auditory target stimuli was analyzed. Peak amplitude and latency were determined at PPOz, POz, and POOz in the time window 450 - 680 ms after target onset.

For the *auditory-visual DFD*, the N1 peak amplitude and latency relative to the auditory distractor was determined for each of the three distractor types in the time-window 80–120 ms after stimulus onset at Fz, FCz, and Cz. The most prominent ERP in this paradigm was the auditory P3 evoked by novel distractors. In order to investigate the novelty-related brain response, difference waves were obtained for every participant. Individual ERPs elicited by the standard distractors were subtracted from those elicited by novel distractors (Escera et al., 1998; Cortiñas et al., 2008; SanMiguel et al., 2008). Differences in peak amplitude and latency were determined at Fz, FCz, and Cz in the time window 200–300 ms (NH) and 250–350 ms (CI), respectively.

In a next step, we analyzed the ERPs elicited by visual targets. Similar to Cortiñas et al., (2008) peak detection was conducted on the P1, N1, and the posterior P3 elicited by the visual targets. This was done separately for all three distractor types (visual stimulus preceded by a standard, deviant or novel distractor). The P1 was identified as the first positive peak in the 80 - 110 ms interval after visual target onset at I1, OI1, OI2, I2 and the N1 was identified as the following negative peak in the same interval. Likewise in the study by Cortiñas and colleagues, the time-window 200–0 ms preceding the auditory distractor was used as baseline (Cortiñas et al., 2008). For amplitude analysis, the P1-N1 peak-to-peak amplitude was calculated for each participant and distractor type. For one out of 12 CI users an identification of the P1/N1 was not possible. For this participant, the P1 and N1 latency as well as the P1-N1 peak-to-peak amplitude was taken as the mean value of the remaining 11 CI users, similar to the procedure in a previous study (Viola et al., 2011). For the detection of the P3 peak amplitude and latency, the maximum positive peak was determined for each individual and distractor type in the time window 360–630 ms (at PPOz, POz, and POOz) relative to the onset of the visual target.

Statistical analyses for the P1, N1, P2, and P3 were carried out using separate repeated-measures ANOVAs for each peak, including Group (NH, CI) as between factor and and Distractor Type (standard, deviant, novel) as within factors. For the novelty-P3/N2 analyses, difference waveforms (novel minus standard) were analyzed using repeated measures ANOVAs with the between factor Group (NH, CI). The above named channels were pooled into regions-of-interest to improve the signal-to-noise ratio (Sandmann et al., 2010). The region-of-interest was computed depending in the ERP component. For the visual-auditory DFD as follows: visual P2 (I1, OI1, OI2, and I2), novelty-N2 (Fz, FCz, and Cz), auditory P3b (PPOz, POz, and POOz). For the auditory-visual DFD as follows: auditory N1 (Fz, FCz, and Cz), novelty-P3 (Fz, FCz, and Cz), visual P1/N1 (I1, OI1, OI2, and I2), visual P3b (PPOz, POz, and POOz). For all statistical analysis SPSS 21 was used. Degrees of freedom (df) were adjusted using a Greenhouse-Geisser correction whenever sphericity was violated. A Bonferroni correction was used for *post-hoc* t-tests in case of multiple testing. Alpha was set at 0.05 for all analyses and partial eta square is reported as a measure for effect size.

## Results

### Performance

**Figure 2:**
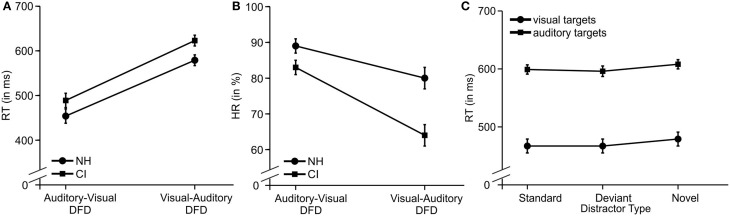
**The behavioral results. (A)** Main effect for Modality and for Group regarding RT. **(B)** Interaction for Group x Modality regarding HR. **(C)** Main effect for Distractor Type on RTs in the auditory-visual DFD (visual targets) and the visual-auditory DFD (auditory targets). Error bars denote the standard error of the mean.

Mean RTs and HRs for the two DFD tasks and the three distractor types are displayed for both CI user and NH participants in Figure 2.

A repeated-measures ANOVA was computed with the between-subjects factor Group (CI, NH) and the within-subjects factors Distractor Type (standard, deviant, novel) and Modality (audio-visual DFD, visual-auditory DFD).

RTs to visual targets were shorter and more accurate compared to RTs to auditory targets for all participants. See Figures 2A,B for the main effects for Modality (RTs: [*F*_(1, 22)_ = 206.157; *p* < 0.001, η^2^_*p*_ = 0.904]; HRs: [*F*_(1, 22)_ = 57.677; *p* < 0.001, η^2^_*p*_ = 0.724]).

CI users responded slower and less accurately compared to NH participants. It is worth noting that this effect occurred in similar strength on both DFD paradigms. See Figures 2A,B for the two main effects of Group (RTs: [*F*_(1, 22)_ = 4.780; *p* = 0.040, η^2^_*p*_ = 0.178]; HRs: [*F*_(1, 22)_ = 8.746; *p* = 0.007, η^2^_*p*_ = 0.284]). Novel distractors prolonged RTs, a finding that replicates the previously described distraction effect (see Figure 2C). *Post-hoc* tests of this main effect of Distractor Type [*F*_(2, 44)_ = 13.996; *p* < 0.001, η^2^_*p*_ = 0.389, corrected] revealed significant differences between novel distractors and standard distractors (*p* = 0.001) or deviant distractors (*p* = 0.003), but no difference was found between standard and deviant distractors (*p* = 1.000).

The interaction Modality x Group for HR reached statistical significance [*F*_(1, 22)_ = 7.587; *p* = 0.012, η^2^_*p*_ = 0.256]. *Post-hoc* tests confirmed the main effect of Modality with lower HRs for auditory than visually presented targets for both groups [CI: *t*_(11)_ = 6.807, *p* < 0.001; NH: *t*_(11)_ = 3.724, *p* = 0.012]. Moreover, HRs were lower in CI users compared to NH for auditory targets [*t*_(22)_ = −3.271, *p* = 0.012] but not for visual targets [*t*_(22)_ = −1.829, *p* = 0.324].

### Event-related potentials

#### Visual-auditory paradigm

**Figure 3:**
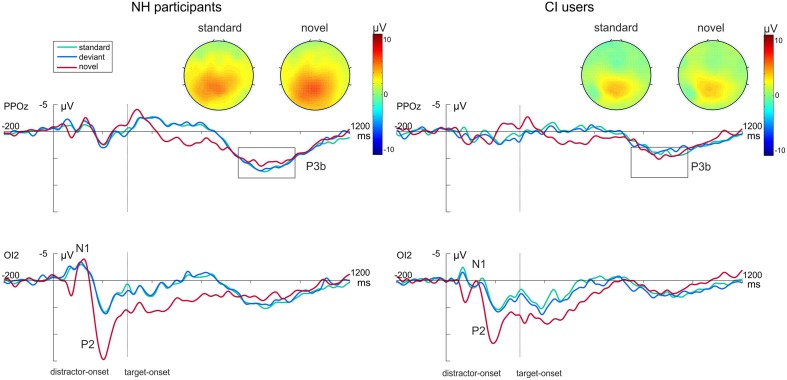
**Grand average ERP data for NH participants (left) and CI users (right) in the visual-auditory DFD at PPOz and IO2**. Standard trials are marked in green, deviant trials in blue and novel trials in red. Onset of the visual task-relevant stimulus is at 300 ms. Please note that for each analyzed component one channel is displayed exemplary. Also the topographical view of the P3b elicited by task-relevant visual stimuli is displayed. See the Result section for more details.

**Figure 4:**
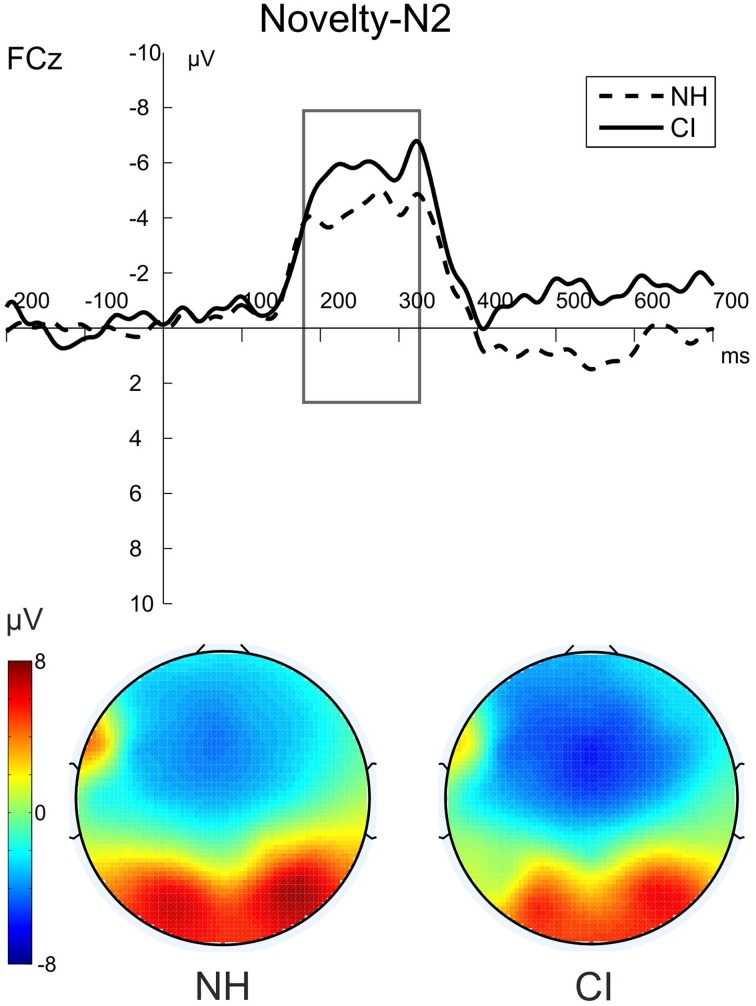
**The novelty-N2 (novel-standard difference waveforms) for NH participants (dashed) and CI users (solid) in the visual-auditory DFD**. The figure shows the ERPs at FCz and the voltage maps illustrating the mean over the analyzed time window (180–330 ms) for NH participants on the left and for CI users on the right.

Figure 3 shows the grand average waveforms evoked by the visual-auditory DFD for all three Distractor Types. Difference waveforms, obtained by subtracting the ERP elicited by standard distractors from those elicited by novel distractors, and the related scalp topographies are shown in Figure 4.

#### Visual distractors

In order to gain insight into the perceptual processing of visual distractors, the P2 amplitude and latency were analyzed by repeated-measures ANOVAs with the between-subjects factor Group (CI, NH) and the within-subjects factor Distractor Type (standard, deviant, novel). See Figure 3 (bottom row) for P2 effects. No main effect for Group was found for this component. The P2 amplitudes were significantly enhanced for novel distractors compared to standard or deviant distractors (main effect for Distractor Type: [*F*_(2, 44)_ = 49.752; *p* < 0.001, η^2^_*p*_ = 0.693, corrected; both *p* < 0.001]). The interaction Distractor Type x Group reached significance level for P2 latencies [*F*_(2, 44)_ = 3.63; *p* = 0.034, η^2^_*p*_ = 0.142]. *Post-hoc* tests uncovered shortened latencies for novel distractors compared to standard distractors in CI users only (*p* = 0.045; Figure 3, bottom row).

Post-perceptual processing of visual distractors was investigated by means of the novelty-N2 (difference waveforms of novel minus standard) and introduced into two repeated-measures ANOVAs with the between factor Group (CI, NH). No differences between CI users and NH participants were found for novelty-N2 amplitude [*F*_(1, 22)_ = 0.385; *p* = 0.541, η^2^_*p*_ = 0.017] or latency [*F*_(1, 22)_ = 0.671; *p* = 0.421, η^2^_*p*_ = 0.030]. See Figure 4.

#### Auditory targets

Additionally, we analyzed post-perceptual processing of auditory targets. No P3 effects were found in the visual-auditory DFD (top row Figure 3). Neither P3 amplitudes [*F*_(1, 22)_ = 0.2.352; *p* = 0.139, η^2^_*p*_ = 0.097] nor P3 latencies [*F*_(1, 22)_ = 2.493; *p* = 0.129, η^2^_*p*_ = 0.102] differed between groups. No significant effect of Distractor Type was found.

#### Auditory-visual paradigm

**Figure 5:**
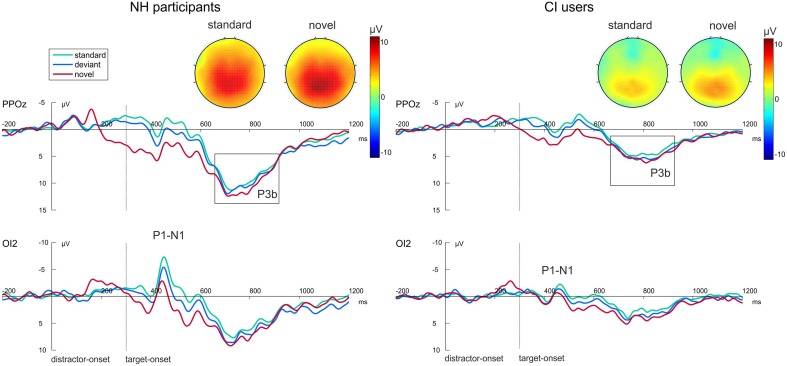
**Grand average ERP data for NH participants (left) and CI users (right) in the auditory-visual DFD at PPOz and IO2**. Standard trials are marked in green, deviant trials in blue and novel trials in red. Onset of the visual task-relevant stimulus is at 300 ms. Please note that for each analyzed component one channel is displayed exemplary. Also the topographical view of the P3b elicited by task-relevant visual stimuli is displayed. See the Result section for more details.

**Figure 6:**
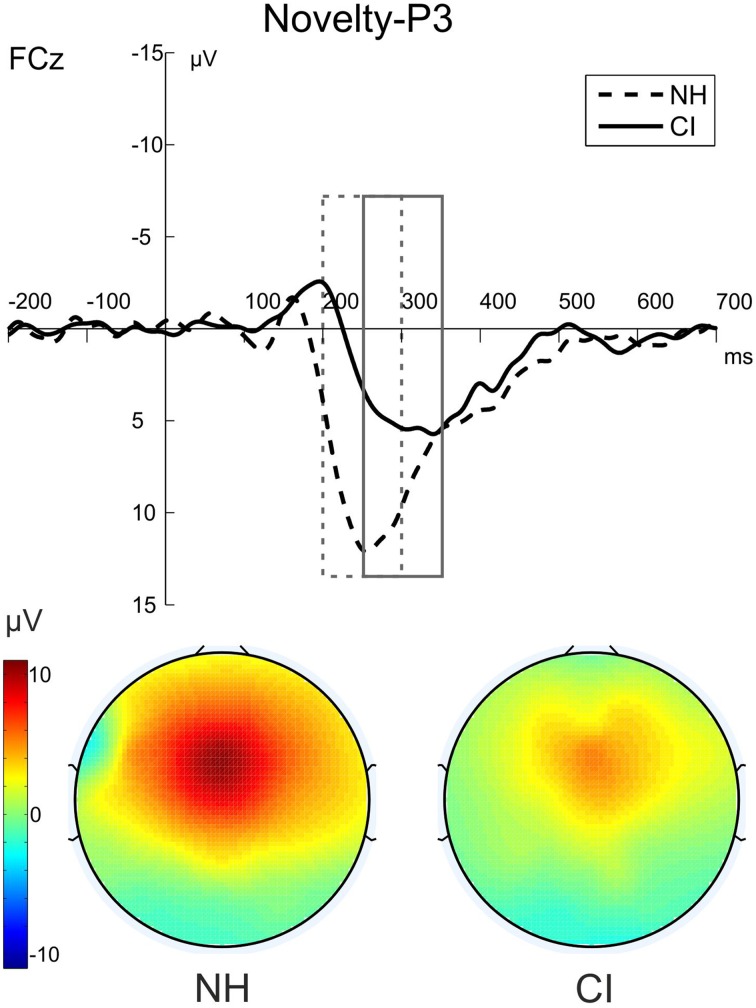
**The novelty-P3 (novel-standard difference waveforms) for NH participants (dashed) and CI users (solid) in the auditory-visual DFD**. The figure shows the ERPs at FCz and the voltage maps illustrating the mean over the analyzed time window for NH participants (200–300 ms) on the left and for CI users (250–350 ms) on the right.

Difference waveforms obtained by subtracting the ERP elicited by standard distractors from those elicited by novel distractors and their topographies across the scalp are shown in Figure 5. Figure 6 shows grand average ERP waveforms that were obtained from the auditory-visual DFD task. These ERP waveforms were evoked in response to auditory distractors followed by visual targets, separately for the three Distractor Types.

#### Auditory distractors

The perceptual processing of auditory distractors was investigated by the means of N1 amplitude and latency. A repeated-measures ANOVA was computed with the between-subjects factor Group (CI, NH) and the within-subjects factor Distractor Type (standard, deviant, novel). The N1 latencies were prolonged in CI users compared to NH participants for all types of stimuli. This resulted in a significant main effect of Group [*F*_(1, 22)_ = 4.329; *p* = 0.049, η^2^_*p*_ = 0.164]. Neither an effect for Distractor Type was found, nor an interaction between the two factors.

The post-perceptual processing of novel distractors was analyzed using the novelty-P3 amplitudes and latencies. Two separate repeated-measures ANOVAs with the between-subjects factor Group (CI, NH) were conducted for novelty-P3 amplitudes and latencies. Novelty-P3 amplitudes were significantly reduced and latencies prolonged in CI users compared to NH participants. See Figure 5 for the two main effects of Group (Amplitude: [*F*_(1, 22)_ = 5.375; *p* = 0.030, η^2^_*p*_ = 0.196]; Latency: [*F*_(1, 22)_ = 15.015; *p* = 0.001, η^2^_*p*_ = 0.406]).

To rule out alternative explanations of the novelty-P3 results, we additionally analyzed the amplitude that resulted from computing the difference waves between standard distractors minus deviant distractors. No difference between groups was found in these difference waves [*F*_(1, 22)_ = 0.057; *p* = 0.814, η^2^_*p*_ = 0.003].

#### Visual targets

The onset of the visual targets elicited clear visual P1 and N1 components at occipital electrodes (see Figure 6, bottom row). Latencies of these early components as well as the P1-N1 peak-to-peak amplitude were analyzed using separate repeated-measures ANOVAs with the between-subjects factor Group (CI, NH) and the within-subjects factor Distractor Type (standard, deviant, novel). The P1-N1 peak to peak amplitude was significantly reduced in CI users compared to NH participants as indicated by the main effect for Group [*F*_(1, 22)_ = 8.800; *p* = 0.007, η^2^_*p*_ = 0.286]. No effects were found on the P1 latencies. N1 latencies did not differ between groups. However, they were significantly shorter after novel distractors compared to standard (*p* < 0.001) or deviant sounds (*p* < 0.001), as indicated by a main effect of Distractor Type [*F*_(2, 44)_ = 22.622; *p* < 0.001, η^2^_*p*_ = 0.507].

Importantly, we also examined post-perceptual processing of visual targets. See Figure 6 for P3 effects. Two separate repeated-measures ANOVAs with the between factor Group (CI, NH) and the within factor Distractor Type (standard, deviant, novel) were conducted for P3 amplitudes and latencies. P3 amplitudes in the NH group were larger than in the CI group as indicated by a main effect of Group [*F*_(1,22)_ = 9.410; *p* = 0.006, η^2^_*p*_ = 0.300]. Moreover, the P3 amplitude revealed a main effect for Distractor Type [*F*_(2, 44)_ = 10.241; *p* < 0.001, η^2^_*p*_ = 0.318]. *Post-hoc* tests uncovered that P3 amplitudes were enhanced for target stimuli following novel distractors compared to target stimuli following standard distractors (*p* = 0.001). The two factors did not show a significant interaction. No effects regarding the P3 latency were found.

## Discussion

The concurrent recording of EEG and performance during the two DFD paradigms enabled us to investigate (1) the orienting response elicited by visual distractors and their influence on the processing of an auditory discrimination task (visual-auditory DFD), (2) the orienting response elicited by auditory distractors and their influence on the processing of a visual task (auditory-visual DFD), and (3) the perceptual processing of auditory and visual stimuli when they occurred either as a distractor or as a target on the two DFD paradigms. In the auditory-visual DFD, we observed differences between CI users and NH individuals with regard to response times and neural indices of post-perceptual processing of auditory distractors and visual targets. Specifically, auditory novelty-P3 amplitudes as well as visual P3 amplitudes were significantly reduced and auditory novelty-P3 latencies were prolonged in CI users compared to NH participants. In the visual-auditory DFD, however, we did not find group differences with regard to neural indices of post-perceptual processing of visual distractors or auditory targets. Our results suggest that the spectro-temporally degraded CI input receives prioritized access to capacity-limited attentional resources in the auditory-visual DFD. Thus, we propose that auditory CI input diminishes cortical processing of subsequent attentional demands in an amodal manner.

### Distraction effects in the visual-auditory DFD

Visual novel distractors prolonged RTs compared to standard or deviant distractors in auditory-visual DFD tasks. This replicates the distraction effect that has repeatedly been reported to occur in response to visual distractors (Schröger and Wolff, 1998; Escera et al., 2000; Berti and Schröger, 2001; Schomaker and Meeter, 2014). As expected, the accuracy for auditory targets in the visual-auditory DFD was reduced in CI users when compared with NH listeners. Poorer auditory performance in CI users can be explained by the degradation of the speech signal through the CI which makes speech understanding more difficult in CI users than in NH listeners. Apart from technical limitations of the CI, physiological changes, among them the deterioration of spiral ganglion cells and the shrinkage of cell bodies, might limit speech intelligibility in CI users (Nadol, 1997). On the one hand, these physiological changes may cause profound hearing loss. On the other hand, they are caused by the hearing loss or deafness (Drennan and Rubinstein, 2008; Sandmann et al., 2012, 2015).

Our ERP data showed the occurrence of a visual novelty-N2 in the auditory-visual DFD. This finding replicates previous work (Schomaker and Meeter, 2014). In agreement with our hypothesis, the two groups showed indistinguishable visual novelty-N2 amplitudes. We conclude from this result that both groups processed visual novelty in similar ways. Further, the P3 component elicited by auditory targets did not differ between groups. In sum, these ERP data suggest that post-perceptual processing of visual distractors and auditory targets did not differ between the two groups. Specifically, they suggest that the distribution of attentional resources to task-irrelevant novel stimuli and to task-relevant auditory stimuli was similar in CI users and in NH participants.

The result according to which P3 amplitudes were indistinguishable in the two groups can be related to the fact that the used stimulus set of one-digit numbers was small and well-known to the participants (8 different numbers ranging from 2 to 9). Moreover, speech intelligibility in the HSM sentence test without noise was excellent in our sample of CI users. Thus, neural processing of auditory targets does not seem to differ between NH participants and CI users who perform at good or very good levels of speech intelligibility. However, we speculate that CI users with low speech intelligibility might have more difficulties to perform our auditory discrimination task. Thus, it remains a possibility that auditory P3 amplitudes could be attenuated specifically in CI users who demonstrate poor speech recognition abilities.

### Distraction effects in the auditory-visual DFD

Auditory novel distractors prolonged RTs compared to standard or deviant distractors, replicating the results from earlier studies (Escera et al., 1998; Cortiñas et al., 2008; SanMiguel et al., 2008). Interestingly, we found a main effect of group regarding RTs for visual targets: CI users responded slower (and less accurately) than normal hearing participants. The result of a group difference for the discrimination ability of *visual* targets might appear surprising. However, there are two potential explanations for this effect. On the one hand, the slowed RTs to visual targets could be explained by neural modifications as a consequence of auditory deprivation and subsequent cochlear implantation. Specifically, there is increasing evidence for cross-modal reorganization in CI users (Giraud et al., 2001a), that is, a visual take-over in the auditory cortex of CI users, to compensate for the loss of auditory input (Doucet et al., 2006; Buckley and Tobey, 2010; Sandmann et al., 2012). On the other hand, our result of prolonged RTs in CI users could be due to a resource capture induced by auditory input. Consequently, fewer resources remain for the processing of the visual task. We suggest that this resource capture occurs specifically in response to auditory distraction in CI users.

In the auditory-visual DFD we expected attenuated post-perceptual ERP waves whenever CI input has to be processed. Consistent with our hypothesis, the auditory novelty-P3 was diminished and delayed in CI users. First, this replicated the results from previous studies (Nager et al., 2007; Torppa et al., 2012). Second, according to our task design, the smaller and delayed novelty-P3 peaks suggest an altered orienting response toward auditory distractors in CI users. In the two aforementioned studies, participants were watching movies rather than performing discrimination tasks. Hence, these studies could not distinguish the contribution of bottom-up (sensory) and top-down (attentional) effects on their novelty-P3 findings in CI users. In an attempt to distinguish between bottom-up (sensory) and top-down (attentional) effects on our novelty-P3 findings in CI users, we analyzed the difference wave between deviant and standard ERPs. If pure bottom-up (sensory) effects accounted for attenuated novelty-P3 amplitudes, we would also have found differences in this analysis, and this was clearly not the case. Taken together, we propose that the present novelty-P3 effects in CI users are related to top-down processes. Similarly, previous studies related modulations in the novelty-P3 to altered attentional orienting responses as well as WM processes (Cortiñas et al., 2008; SanMiguel et al., 2008).

We analyzed the ERPs elicited by visual targets in order to test our hypothesis that CI input might be associated with an additional load on the capacity-limited attentional resource. Visual P3 amplitudes were attenuated in the group of CI users compared to the NH group. According to previous interpretations of P3 reductions in the DFD paradigm, this attenuation suggests that attentional resources for post-perceptual processing of task-relevant stimuli are reduced in the CI users (Alho et al., 1997; Cortiñas et al., 2008). Importantly, visual P3 amplitudes were diminished in CI users irrespective of the type of the auditory distractor that preceded a visual target. Similar results have been found by Cortiñas et al. (2008). They reasoned that the effects on P3 and RT result due to allocation of attention toward the distractors (although, they reported on schizophrenic patients and not CI users).

Moreover, other studies support our interpretation of the P3 data according to which the attenuated visual P3 amplitudes in CI users indicate that attentional resources for post-perceptual processing of task-relevant stimuli are reduced in temporal vicinity of the processing of CI input, even though these studies made use of a slightly different approach. The limited availability of central resources was investigated with so-called attentional blink paradigm. Vogel et al. (1998) recorded ERPs in order to determine whether the visual AB reflects suppressed perceptual processes or impaired post-perceptual processes. A complete suppression in response to the second (undetected) target during the AB interval was found for the P3 component but not for the N1 component. This suggests that post-perceptual resources might have been be allocated to the processing of T1 resulting in a failure to detect T2. The finding that T2-P3 suppression does also occur on cross-modal AB paradigms supports the idea that the AB is caused by the depletion of a central attentional resource (Dell'acqua et al., 2003; Arnell, 2006; Ptito et al., 2008). Our results resemble the results obtained in single- and dual-task AB studies (Vogel et al., 1998; Dell'acqua et al., 2003; Brisson and Jolicœur, 2007). The P3 amplitudes elicited by T2 decreased in these AB paradigms with increasing demands on the central attentional resource for T1. This can be induced by manipulating temporal lags between T1 and T2 or by increasing task difficulty. The similarity between the AB and our DFD paradigms lies in the fact that distractors (T1) and targets (T2) are presented in close temporal vicinity. Viewed from this perspective, the visual P3 amplitude attenuation in CI users indicates that distractors transmitted via CIs (equivalent to T1-processing) captured attention at the expense of visual target (T2) processing in the auditory-visual DFD task. We thus report here for the first time that the distribution of attentional resources to task-irrelevant auditory stimuli and to task-relevant visual stimuli is altered in CI users. Specifically, CI-induced auditory processing seems to interfere with subsequent visual processing, putatively via placing additional loads on central attentional resources.

### CI-induced resource capture model (CIRC)

We suggest that the processing of CI-input is associated with an additional load on capacity-limited attentional resources.

Based on our results and evidence from the literature, we propose a CI-induced resource capturing (CIRC) model. It proposes that the processing of auditory CI-input captures additional attentional resources. According to Kahneman (1973), the central resources are strictly capacity-limited (Kahneman, 1973; Vogel et al., 1998). Consequently, less attentional resources remain available for the processing of other input in temporal vicinity of CI input. The effects of CI-induced resource capturing could be mediated by inter-individual differences in attentional capacity. However, when the remaining attentional resources fall below a certain threshold, indices of task performance start to decline.

Kahneman (1973) described in his model attentional capacity as one of the key components to solve a task successfully. This component in turn depends on a certain amount of arousal and allocation of attention. Kahneman assumed that focusing on the target and ignoring distractors allows appropriate allocation of attentional resources (Kahneman, 1973). The DFD paradigms nicely simulate this idea in the laboratory.

The participants' situation of being asked to perform a visual discrimination task while being exposed to task-irrelevant changing sounds resembles a situation like reading a book in a noisy environment (i.e., in the train or in a coffee bar). We base our CIRC model on Kahneman's traditional psychological model regarding attentional resources and on the large evidence of P3 studies. Based on the broad P3 literature we assume that our novelty-P3 and P3 effects reflect resource capturing through additional allocation of attention to CI-input (Escera et al., 2000; Kok, 2001; Brisson and Jolicœur, 2007; Polich, 2007; SanMiguel et al., 2008).

It should be mentioned that the ELU model (Rönnberg et al., 2013) and the CIRC model are not mutually exclusive. We assume that CI input yields extremely suboptimal stimuli for listening. As a consequence, CI users need more attentional resources to process auditory inputs under all circumstances. In contrast, NH listeners process only explicitly when there is a mismatch between actual input and patterns stored in memory (like in adverse listening conditions). We found P3 amplitudes to be reduced in CI users subsequently to all types of auditory distractors (novel, deviant, and standard distractors). As expected, P3 amplitude attenuation did not only follow auditory novel distractors but also standard and deviant distractors. This result supports the idea that resource capture in CI users is a phenomenon unrelated to novelty but results from any distractor (Figure 5). This suggests a global resource capture induced by any auditory CI input. The ELU model proposes that when listening conditions are adverse, additional (attentional) processing is needed to understand speech. We interpret our data in the following way: When auditory input via a CI is processed (and listening conditions are therefore adverse), additional resources are needed to process the auditory input. If stimuli are presented in temporal vicinity, we find reduced P3 amplitudes and slowed RTs; a similar effect as in AB paradigms and probably due to a lack of attentional resources. The CIRC model, like the ELU model proposes that more resources are needed when listening in adverse conditions. We additionally suggest that in rapid stimulus presentation, the attentional resources allocated to auditory input are unavailable to process a task in temporal vicinity.

### Perceptual processing of stimuli

Although not the focus of the present study, we analyzed the ERPs reflecting the perceptual processing of auditory and visual stimuli. Depending on the DFD task, they were either a distractor or a target. In the auditory-visual DFD, we found delayed auditory N1 latencies in CI users compared to NH participants. Longer N1 latencies in CI users compared to NH participants have been found in previous studies (Timm et al., 2012; Sandmann et al., 2015). Cortical adaption to the CI signal during the first year after implantation has been shown in a recent longitudinal study (Sandmann et al., 2015). Nonetheless N1 latencies and amplitudes in CI users showed remarkable changes in the first year after implantation, differences to NH participants persisted. CI users of the present study used their implant for at least 1 year, quite a few for several years. This goes in line with the assumptions that (1) the most prominent adaptation to the CI occur in the first months after implantation and (2) even long-term CI users do show differences in auditory-evoked potentials when compared with NH listeners (Sandmann et al., 2009, 2010, 2015; Timm et al., 2012).

Moreover we found differences in the visual-evoked potentials (VEP) between CI users and NH participants. The visual P1-N1 peak-to-peak amplitudes elicited by targets in the auditory-visual DFD were reduced in CI users. Regarding the visual-auditory DFD, the P2 latencies were shorter for novel distractors compared to standard distractors in CI users, while such a condition effect was not present in the group of NH listeners. These results are in line with previous observations that CI users and NH listeners show differences in auditory as well as visual cortex functions (Sandmann et al., 2012). Likewise, previous longitudinal studies have revealed that neuroplasticity after cochlear implantation involves not only auditory but also visual and audiovisual cortical networks (Giraud et al., 2001b; Strelnikov et al., 2009; Rouger et al., 2012). Furthermore, there is increasing evidence for a visual-to-auditory cross-modal reorganization in the auditory cortex of CI users (Lee et al., 2001). In sum, these results suggest that long-term alteration of auditory experience (or the lack thereof) induce functional changes that are not limited to the auditory cortex but also extend to the visual cortex.

## Summary and conclusions

In summary, the present study revealed initial evidence that the allocation of attentional resources in CI users is altered in an auditory-visual DFD. Importantly, we do not presume that attentional resources are generally reduced in CI users. Our results suggest that when CI users are exposed to auditory distractors, these individuals need additional attentional resources to process the auditory input, at the expense of processing stimuli in temporal vicinity of the CI-input. Additional attentional resources are needed to process any type of auditory input, not only novel auditory input. In contrast, NH seem to need an additional allocation of attention only in adverse auditory listening conditions (Zekveld et al., 2006; Obleser et al., 2012; Rönnberg et al., 2013).

Finally, we suggest that one might consider CI-induced resource capturing when arranging ergonomics at work places for CI users in everyday life. Noise reduction and quiet environments (e.g., no open plan office) are possibilities which would facilitate CI users to better focus on work-relevant (visual) tasks and to reduce fatigue. Furthermore, our results suggest that there is a need to implement specific exercises to train attentional allocation and cognitive multi-tasking in CI rehabilitation.

### Conflict of interest statement

The authors declare that the research was conducted in the absence of any commercial or financial relationships that could be construed as a potential conflict of interest.

## References

[B1] Alho K. Escera C. Díaz R. Yago E. Serra J. M. (1997). Effects of involuntary auditory attention on visual task performance and brain activity. Neuroreport 8, 3233–3237. 10.1097/00001756-199710200-00010 9351649

[B1a] Arnell K. M. (2006). Visual, auditory, and cross-modality dual-task costs: electrophysiological evidence for an amodal bottleneck on working memory consolidation. Percept. Psychophys. 68, 447–457. 10.3758/BF03193689 16900836

[B2a] Berti S. Schröger E. (2001). A comparison of auditory and visual distraction effects: behavioral and event-related indices. Cogn. Brain Res. 10, 265–273. 10.1016/S0926-6410(00)00044-6 11167050

[B3a] Buckley K. A. Tobey E. A. (2010). Cross-modal plasticity and speech perception in pre- and postlingually deaf cochlear implant users. Ear Hear. 32, 2–15. 10.1097/AUD.0b013e3181e8534c 20829699

[B2] Brisson B. Jolicœur P. (2007). Cross-modal multitasking processing deficits prior to the central bottleneck revealed by event-related potentials. Neuropsychologia 45, 3038–3053. 10.1016/j.neuropsychologia.2007.05.022 17659310

[B3] Broadbent D. (1958). Perception and Communication. London: Pergamon Press.

[B4] Bunzeck N. Doeller C. F. Dolan R. J. Duzel E. (2012). Contextual interaction between novelty and reward processing within the mesolimbic system. Hum. Brain Mapp. 33, 1309–1324. 10.1002/hbm.21288 21520353 PMC3498733

[B5] Bunzeck N. Guitart-Masip M. Dolan R. J. Duzel E. (2014). Pharmacological dissociation of novelty responses in the human brain. Cereb. Cortex 24, 1351–1360. 10.1093/cercor/bhs420 23307638 PMC3977623

[B6] Cherry E. C. (1953). Some experiments on the recognition of speech with one and with two ears. J. Acoust. Soc. Am. 25, 975–979 10.1121/1.1907229

[B7] Cortiñas M. Corral M.-J. Garrido G. Garolera M. Pajares M. Escera C. (2008). Reduced novelty-P3 associated with increased behavioral distractibility in schizophrenia. Biol. Psychol. 78, 253–260. 10.1016/j.biopsycho.2008.03.011 18450358

[B8] Dell'acqua R. Jolicoeur P. Pesciarelli F. Job R. Palomba D. (2003). Electrophysiological evidence of visual encoding deficits in a cross-modal attentional blink paradigm. Psychophysiology 40, 629–639. 10.1111/1469-8986.00064 14570170

[B9] Deutsch J. Deutsch D. (1963). Attention: some theoretical considerations. Psychol. Rev. 70, 80–90. 10.1037/h0039515 14027390

[B10] Dien J. (2010). The ERP PCA Toolkit: an open source program for advanced statistical analysis of event-related potential data. J. Neurosci. Methods 187, 138–145. 10.1016/j.jneumeth.2009.12.009 20035787

[B12] Donchin E. Coles M. G. H. (1988). Is the P300 component a manifastation of context updating? Behav. Brain Sci. 11, 357–374. 10.1017/S0140525X0005802722974337

[B13] Donchin E. Coles M. G. H. (1998). Context Updating and the P300. Behav. Brain Sci. 21, 152–154 10.1017/S0140525X98230950

[B4a] Doucet M. E. Bergeron F. Lassonde M. Ferron P. Lepore F. (2006). Cross-modal reorganization and speech perception in cochlear implant users. Brain 129, 3376–3383. 10.1093/brain/awl264 17003067

[B14] Drennan W. R. Rubinstein J. T. (2008). Music perception in cochlear implant users and its relationship with psychophysical capabilities. J. Rehabil. Res. Dev. 45, 779–789. 10.1682/JRRD.2007.08.0118 18816426 PMC2628814

[B15] Eimer M. Holmes A. (2002). An ERP study on the time course of emotional face processing. Neuroreport 13, 427–431. 10.1097/00001756-200203250-00013 11930154

[B16] Escera C. Alho K. Schröger E. Winkler I. (2000). Involuntary attention and distractibility as evaluated with event-related brain potentials. Audiol. Neurootol. 5, 151–166. 10.1159/000013877 10859410

[B17] Escera C. Alho K. Winkler I. Näätänen R. (1998). Neural mechanisms of involuntary attention to acoustic novelty and change. J. Cogn. Neurosci. 10, 590–604. 10.1162/089892998562997 9802992

[B18] Escera C. Corral M.-J. (2003). The distraction potential (DP), an electrophysiological tracer of involuntary attention control and its dysfunction, in The Cognitive Neuroscience of Individual Differences, eds Reinvang I. Greenlee M. W. Herrmann M. (Oldenburg: Bibliotheks- und Informationssystem der Universität Oldenburg), 63–76.

[B19] Giraud A.-L. Price C. J. Graham J. M. Truy E. Frackowiak R. S. J. (2001a). Cross-Modal plasticity underpins language recovery after cochlear implantation. Neuron 30, 657–664. 10.1016/S0896-6273(01)00318-X 11430800

[B20] Giraud A. L. Truy E. Frackowiak R. (2001b). Imaging plasticity in cochlear implant patients. Audiol. Neurootol. 6, 381–393. 10.1159/000046847 11847465

[B21] Hahlbrock K. H. (1953). Speech audiometry and new word-tests. Arch. Ohren. Nasen. Kehlkopfheilkd. 162, 394–431. 10.1007/BF02105664 13092895

[B24] Hochmair-Desoyer I. Schulz E. Moser L. Schmidt M. (1997). The HSM sentence test as a tool for evaluating the speech understanding in noise of cochlear implant users. Am. J. Otolaryngol. 18, 83. 9391610

[B25] Humes L. E. (2007). The contributions of audibility and cognitive factors to the benefit provided by amplified speech to older adults. J. Am. Acad. Audiol. 18, 590–603. 10.3766/jaaa.18.7.6 18236646

[B26] Kahneman D. (1973). Attention and Effort. Englewood Cliffs, NJ: Prentice-Hill.

[B27] Kanwisher N. McDermott J. Chun M. M. (1997). The fusiform face area: a module in human extrastriate cortex specialized for face perception. J. Neurosci. Off. J. Soc. Neurosci. 17, 4302–4311. 9151747 10.1523/JNEUROSCI.17-11-04302.1997 PMC6573547

[B28] Kok A. (2001). On the utility of P3 amplitude as a measure of processing capacity. Psychophysiology 38, 557–577. 10.1017/S0048577201990559 11352145

[B29] Krueger B. Joseph G. Rost U. Strau-Schier A. Lenarz T. Buechner A. (2008). Performance groups in adult cochlear implant users: speech perception results from 1984 until today. Otol. Neurotol. 29, 509–512. 10.1097/MAO.0b013e318171972f 18520586

[B30] Lee D. S. Lee J. S. Oh S. H. Kim S.-K. Kim J.-W. Chung J.-K.. (2001). Deafness: cross-modal plasticity and cochlear implants. Nature 409, 149–150. 10.1038/35051653 11196628

[B31] Näätänen R. Jacobsen T. Winkler I. (2005). Memory-based or afferent processes in mismatch negativity (MMN): a review of the evidence. Psychophysiology 42, 25–32. 10.1111/j.1469-8986.2005.00256.x 15720578

[B32] Nadol J. B. (1997). Patterns of neural degeneration in the human cochlea and auditory nerve: implications for cochlear implantation. Otolaryngol. Head Neck Surg. 117, 220–228. 10.1016/S0194-5998(97)70178-5 9334769

[B33] Nager W. Münte T. F. Bohrer I. Lenarz T. Dengler R. Möbes J.. (2007). Automatic and attentive processing of sounds in cochlear implant patients–Electrophysiological evidence. Restor. Neurol. Neurosci. 25, 391–396. 17943014

[B34] Obleser J. Wöstmann M. Hellbernd N. Wilsch A. Maess B. (2012). Adverse listening conditions and memory load drive a common alpha oscillatory network. J. Neurosci. 32, 12376–12383. 10.1523/JNEUROSCI.4908-11.2012 22956828 PMC6621258

[B35] Peterson N. R. Pisoni D. B. Miyamoto R. T. (2010). Cochlear implants and spoken language processing abilities: review and assessment of the literature. Restor. Neurol. Neurosci. 28, 237–250. 10.3233/RNN-2010-0535 20404411 PMC2947146

[B36] Pichora-Fuller M. K. (2006). Perceptual effort and apparent cognitive decline: implications for audiologic rehabilitation. Semin. Hear. 27, 284–293 10.1055/s-2006-954855

[B37] Pichora-Fuller M. K. (2008). Use of supportive context by younger and older adult listeners: balancing bottom-up and top-down information processing. Int. J. Audiol. 47, S72–S82. 10.1080/14992020802307404 19012114

[B38] Pichora-Fuller M. K. Singh G. (2006). Effects of age on auditory and cognitive processing: implications for hearing aid fitting and audiologic rehabilitation. Trends Amplif. 10, 29–59. 10.1177/108471380601000103 16528429 PMC4111543

[B5a] Ptito A. Arnell K. Jolicoeur P. Macleod J. (2008). Intramodal and crossmodal processing delays in the attentional blink paradigm revealed by event-related potentials. Psychophysiology 45, 794–803. 10.1111/j.1469-8986.2008.00677.x 18627533

[B39] Polich J. (2007). Updating P300: an integrative theory of P3a and P3b. Clin. Neurophysiol. 118, 2128–2148. 10.1016/j.clinph.2007.04.019 17573239 PMC2715154

[B40] Rönnberg J. Lunner T. Zekveld A. Sörqvist P. Danielsson H. Lyxell B.. (2013). The Ease of Language Understanding (ELU) model: theoretical, empirical, and clinical advances. Front. Syst. Neurosci. 7:31. 10.3389/fnsys.2013.00031 23874273 PMC3710434

[B6a] Raymond J. E. Shapiro K. L. Arnell K. M. (1992). Temporary suppression of visual processing in an RSVP task: an attentional blink? J. Exp. Psychol. Hum. Percept. Perform. 18, 849–860. 10.1037/0096-1523.18.3.849 1500880

[B41] Rouger J. Lagleyre S. Démonet J.-F. Fraysse B. Deguine O. Barone P. (2012). Evolution of crossmodal reorganization of the voice area in cochlear-implanted deaf patients. Hum. Brain Mapp. 33, 1929–1940. 10.1002/hbm.21331 21557388 PMC6870380

[B7a] Sandmann P. Dillier N. Eichele T. Meyer M. Kegel A. Pascual-Marqui R. D.. (2012). Visual activation of auditory cortex reflects maladaptive plasticity in cochlear implant users. Brain 135, 555–568. 10.1093/brain/awr329 22232592

[B8a] Sandmann P. Eichele T. Buechler M. Debener S. Jäncke L. Dillier N.. (2009). Evaluation of evoked potentials to dyadic tones after cochlear implantation. Brain 132, 1967–1979. 10.1093/brain/awp034 19293240

[B42] Sandmann P. Kegel A. Eichele T. Dillier N. Lai W. Bendixen A.. (2010). Neurophysiological evidence of impaired musical sound perception in cochlear-implant users. Clin. Neurophysiol. 121, 2070–2082. 10.1016/j.clinph.2010.04.032 20570555

[B9a] Sandmann P. Plotz K. Hauthal N. de Vos M. Schönfeld R. Debener S. (2015). Rapid bilateral improvement in auditory cortex activity in postlingually deafened adults following cochlear implantation. Clin. Neurophysiol. 126, 594–607. 10.1016/j.clinph.2014.06.029 25065298

[B43] SanMiguel I. Corral M.-J. Escera C. (2008). When loading working memory reduces distraction: behavioral and electrophysiological evidence from an auditory-visual distraction paradigm. J. Cogn. Neurosci. 20, 1131–1145. 10.1162/jocn.2008.20078 18284343

[B44] Schomaker J. Meeter M. (2014). Novelty detection is enhanced when attention is otherwise engaged: an event-related potential study. Exp. Brain Res. 232, 995–1011. 10.1007/s00221-013-3811-y 24402203

[B10a] Schröger E. Wolff C. (1998). Behavioral and electrophysiological effects of task irrelevant sound change: a new distraction paradigm. Cogn. Brain Res. 7, 71–87. 10.1016/S0926-6410(98)00013-5 9714745

[B45] Semlitsch H. V. Anderer P. Schuster P. Presslich O. (1986). A solution for reliable and valid reduction of ocular artifacts, applied to the P300 ERP. Psychophysiology 23, 695–703. 10.1111/j.1469-8986.1986.tb00696.x 3823345

[B46] Strelnikov K. Rouger J. Barone P. Deguine O. (2009). Role of speechreading in audiovisual interactions during the recovery of speech comprehension in deaf adults with cochlear implants. Scand. J. Psychol. 50, 437–444. 10.1111/j.1467-9450.2009.00741.x 19778391

[B47] Ter Braack E. M. De Jonge B. Van Putten M. J. A. M. (2013). Reduction of TMS induced artifacts in EEG using principal component analysis. IEEE Trans. Neural Syst. Rehabil. Eng. Publ. 21, 376–382. 10.1109/TNSRE.2012.2228674 23359012

[B11a] Timm L. Agrawal D. Viola F. C. Sandmann P. Debener S. Wittfoth M. (2012). Temporal feature perception in cochlear implant users. PLoS ONE 7:e45375. 10.1371/journal.pone.0045375 23028971 PMC3448664

[B48] Torppa R. Salo E. Makkonen T. Loimo H. Pykäläinen J. Lipsanen J.. (2012). Cortical processing of musical sounds in children with Cochlear Implants. Clin. Neurophysiol. 123, 1966–1979. 10.1016/j.clinph.2012.03.008 22554786

[B49] Treisman A. (1964). Selective attention in man. Br. Med. Bull. 20, 12–16. 14104089 10.1093/oxfordjournals.bmb.a070274

[B50] Verleger R. (1997). On the utility of P3 latency as an index of mental chronometry. Psychophysiology 34, 131–156. 10.1111/j.1469-8986.1997.tb02125.x 9090263

[B51] Verleger R. (2008). P3b: towards some decision about memory. Clin. Neurophysiol. 119, 968–970. 10.1016/j.clinph.2007.11.175 18222107

[B52] Viola F. C. Thorne J. D. Bleeck S. Eyles J. Debener S. (2011). Uncovering auditory evoked potentials from cochlear implant users with independent component analysis. Psychophysiology 48, 1470–1480. 10.1111/j.1469-8986.2011.01224.x 21635266

[B53] Vogel E. K. Luck S. J. Shapiro K. L. (1998). Electrophysiological evidence for a postperceptual locus of suppression during the attentional blink. J. Exp. Psychol. Hum. Percept. Perform. 24, 1656–1674. 10.1037/0096-1523.24.6.1656 9861716

[B54] Willenbockel V. Sadr J. Fiset D. Horne G. O. Gosselin F. Tanaka J. W. (2010). Controlling low-level image properties: the SHINE toolbox. Behav. Res. Methods 42, 671–684. 10.3758/BRM.42.3.671 20805589

[B55] Wilson B. S. Dorman M. F. (2008). Cochlear implants: a remarkable past and a brilliant future. Hear. Res. 242, 3–21. 10.1016/j.heares.2008.06.005 18616994 PMC3707130

[B56] Zekveld A. A. Heslenfeld D. J. Festen J. M. Schoonhoven R. (2006). Top–down and bottom–up processes in speech comprehension. Neuroimage 32, 1826–1836. 10.1016/j.neuroimage.2006.04.199 16781167

